# Long-term Satisfaction with CO_2_ Laser Treatment for Moderate to Major Rhinophyma: A Single-centre Study

**DOI:** 10.2340/actadv.v105.41335

**Published:** 2025-01-03

**Authors:** Berta OLAFSDOTTIR, William JEBRIL, Philip CURMAN, Etty BACHAR-WIKSTRÖM, Jakob D. WIKSTRÖM

**Affiliations:** 1 Dermatology and Venereology Division, Department of Medicine (Solna), Karolinska Institutet, Stockholm; 2 Dermato-Venereology Clinic, Karolinska University Hospital, Stockholm; 3 Department of Medical Epidemiology and Biostatistics, Karolinska Institutet, Stockholm; 4 Nordiska kliniken, Stockholm, Sweden, Sweden

**Keywords:** CO_2_ laser, cosmetic dermatology, rhinophyma, rosacea, phymatous, surgical dermatology

## Abstract

Rhinophyma, a severe manifestation of rosacea, predominantly affects Caucasian males aged 50–70 and is characterized by thickening and enlargement of the nasal skin. The condition can seriously both impact cosmetic appearance and obstruct nasal breathing. While its appearance is distinct, conditions such as basal cell carcinoma can mimic it, complicating diagnosis. Treatment options for rhinophyma range from medical therapies to surgical interventions, with CO_2_ laser resurfacing emerging as the gold standard due to its precision and minimal blood loss. This retrospective case series evaluates the long-term efficacy and patient satisfaction of CO_2_ laser treatment in 11 male patients treated between 2015 and 2023 at the Karolinska University Hospital. The results revealed sustained positive outcomes, with 92% of patients reporting high satisfaction and 55% experiencing improved nasal airflow post-procedure. Despite some instances of hypopigmentation and scarring, no major side effects were reported. The study supports CO_2_ laser treatment as a safe and effective option for moderate to major rhinophyma in Caucasian male patients with Fitzpatrick skin types I–III. The importance of considering skin type, where extra caution must be employed when treating skin types IV–VI, should be emphasized as the treatment can lead to considerable hypopigmentation.

## SIGNIFICANCE

This study is important because it sheds light on how CO2 laser treatment can effectively and safely improve rhinophyma, a condition that causes noticeable changes to the nose and can make breathing difficult. The research shows that most patients are happy with their results and even breathe easier after the treatment. It also highlights the importance of considering individual differences, such as skin type and breathing issues, when deciding on this treatment. This information can help both doctors and patients make better decisions on managing rhinophyma.

Rhinophyma is a rare disfiguring nose condition that predominantly affects Caucasian males between 50 and 70 years old (1). It is a more severe form of the inflammatory skin condition rosacea, which is a common chronic skin disorder with a prevalence of 5.5% and is more common in women than in men (2). However, the estimated male-to-female ratio of patients with rhinophyma ranges from 5:1 to 30:1 and is believed to be mediated by increased androgen activity in males (3).

Rosacea is divided into 4 types depending on its severity and clinical manifestations. Type 1 entails vascular rosacea, characterized by flushing and vasodilation leading to permanent erythema and telangiectasia, usually on the central area of the face. Type 2, known as inflammatory rosacea, is caused by persistent or episodic development of inflammatory papules and pustules. Type 3, phymatous rosacea, results in skin hyperplasia caused by chronic inflammation. The usual affected area is the nose, leading to a disfiguration of the nose called rhinophyma. Lastly, type 4, ocular rosacea, is usually characterized by blepharitis and conjunctivitis (4).

Rhinophyma is caused by thickening of the skin and enlargement of its sebaceous glands, resulting in irregular nodular growth. The deformity of the nose can also result in nasal outflow obstruction, which can result in obstructive sleep apnoea (5, 6). Available treatments for rhinophyma include medical treatments such as retinoids and tetracyclines, which can reduce a modest rhinophyma (1). Surgical treatments include partial- or full-thickness excision with or without skin grafting or flap reconstruction, dermaplaning and dermabrasion, cryosurgery, and excision with heated scalpels and loops, electrosurgery, and ultrasonic scalpel. Laser options include carbon dioxide (CO_2_) laser, erbium: yttrium–aluminium–garnet, and argon lasers (5).

No long-term studies have been performed to recommend one treatment over another, as most reports are not powered to generate generalizable conclusions (5, 7). CO_2_ resurfacing has, however, been considered the gold standard laser therapy for treating rhinophyma, as it allows precise sculpting in a bloodless field, but may result in a shiny and scarred nose with patulous pores and hypopigmentation (1, 5, 7). It is considered a relatively safe and effective treatment in the short term, but the long-term outcome and patient satisfaction with this treatment are largely unknown (5, 7).

This retrospective case series aims to evaluate the long-term effects of CO_2_ laser treatment for rhinophyma and evaluate patients’ satisfaction.

## MATERIALS AND METHODS

### Ethical considerations

As a case series, this study is exempt from ethical review according to Swedish legislation (8), which also mandates that the quality of healthcare services will be systematically and continuously developed and ensured. The patients in this manuscript have given written informed consent to the publication of their case details.

### Study subjects

We retrospectively reviewed all patients who underwent CO_2_ laser treatment for rhinophyma at the Department of Dermatology at Karolinska University Hospital from 2015 to 2023.

The inclusion criterion was a diagnosis of rhinophyma that had been treated with a CO_2_ laser at the department. Exclusion criteria were unwillingness or inability to complete the final photo documentation. Eleven men aged 54 to 79 suffering from rhinophyma who had undergone laser treatment from 2015 to 2023 were included. The severity of rhinophyma was based on the classification proposed by el-Azhary et al. (9). According to this classification, patients were categorized as having minor rhinophyma if they exhibited telangiectasia and mild thickening or changes in skin texture. Those with skin thickening and lobules were classified as having moderate rhinophyma. Patients who displayed nasal hypertrophy along with prominent lobules were classified as having major rhinophyma.

### Clinical evaluations

All patients underwent clinical assessment prior to CO_2_ laser treatment and informed consent was obtained. Digital photos taken by a professional photographer were used to evaluate treatment results subjectively. All patients photo-documented their noses before the laser treatment and 3–6 weeks afterwards, except for 1 patient who photo-documented their noses 8 months after receiving the treatment. Lastly, all patients took a third photo in 2024. The authors used the photos to determine short-term and long-term positive effects vs possible side effects.

### Patients questionnaire

All patients answered a questionnaire to assess their long-term satisfaction and response to CO_2_ laser treatment (**Table I**).

**Table I:** Questionnaire answered by patients included in the study

Self-Assessment Questionnaire
I: On a scale of 0–10, how satisfied are you with the treatment (0, very dissatisfied; 10, very satisfied)? 0–10 II: Are the beneficial effects of the laser treatment still maintained? Yes No III: Has the treatment made breathing through the nose easier? Yes No IV: Did you experience any side effects of the treatment? Free text V: Would you recommend this treatment to other patients with rhinophyma? Yes Not sure No VI: Any additional comments? Free text

*Laser treatment*. A CO_2_ laser was used after injections of lidocaine 1% with epinephrine (1:100,000) into the affected area. All personnel and the patient wore laser-protective glasses. A vacuum suction device was used to clear the smoke, which consisted of partly evaporated tissue. All patients were treated in a single session with either a Limmer Laser (UNILAS Touch, Limmer Laser GmbH, Germany) or a newer DEKA laser (DEKA M.E.L.A. S.r.l., Calenzano, Italy). The scanner-assisted pulsed carbon dioxide lasers were used to cause evaporation of excess tissue and defoliate the swollen nasal tissue while preserving follicles. For the Limmer laser, the following settings were used: 2–4 mm diameter spot-size, effect 25–50 W, and 0.3 ms pauses in continuous mode. For the DEKA laser, the following settings were used: 10–25 W, continuous wave, spot size 2–4 mm, dwell time 0.1 ms, aiming 100% repeat off, and a 10-s exposure mode. After the laser treatment, the nose was bandaged with resorbable haemostasis gelatine (Spongostan), which was removed the day after, followed by applying petroleum jelly (Vaseline) daily until fully healed.

## RESULTS

### Clinical results

**Fig 1:**
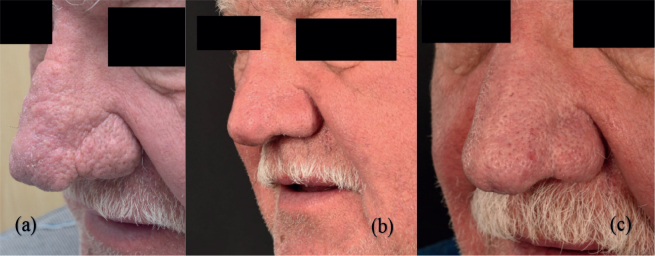
(A) Major rhinophyma with prominent lobules before laser treatment. (B) A significantly better profile 6 weeks postoperatively and (C) 8 months after receiving the treatment.

*Case 1.* A 79-year-old man who was suffering from major rhinophyma. He had previously received laser treatment 15–20 years earlier. He received his second laser treatment in 2023. No complications were reported (**Fig. 1**).

**Fig. 2:**
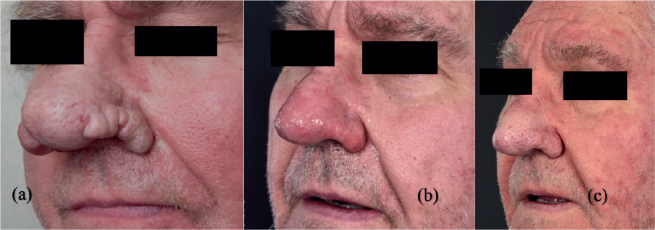
(A) Major rhinophyma before laser treatment, and (B) 2 weeks postoperatively, revealing an improved profile and minimal scarring. (C) Slight hypopigmentation and scarring can be seen 4 years later with a preserved nasal profile.

*Case 2.* A 70-year-old male presented with major rhinophyma (**Fig. 2**). His past medical history included medical treatment with isotretinoin during 3 different periods, and he stopped isotretinoin 6 months prior to receiving the laser treatment. He received laser treatment in 2020 without any immediate complications. After undergoing CO_2_ laser therapy, 10 days postoperatively he suffered from bilateral nosebleeds, which caused him to be referred from primary care to emergency care because the bleeding source could not be located. He was stable but did suffer a brief loss of consciousness and was taken by ambulance to the emergency department, where a well-defined bleeding source could be located on the Kiesselbach plexus on the right side, likely not caused by the laser treatment due to its interior location. No other bleeding source could be located. The bleeding artery was cauterized, which in turn stopped the bleeding. The patient was sent home and had no further complications.

*Case 3.* A 75-year-old man who was suffering from moderate rhinophyma. Immediately after the procedure, a couple of bleeding spots were sutured with 4:0 resorbable sutures (Vicryl). No complications were reported (Fig. S1).

*Case 4.* A 73-year-old man who suffered from moderate rhinophyma. During the procedure, 1 bleeding area centrally on the nose was sutured with resorbable sutures (Vicryl Rapid). No complications were reported (Fig. S2).

*Case 5.* A 70-year-old man presenting with major rhinophyma. He suffered no immediate or late complications (Fig. S3).

*Case 6.* A 67-year-old man received laser treatment for moderate rhinophyma without any postoperative complications (Fig. S4). He had suffered from rhinophyma for a little over a year and had had no positive effects of tetracycline antibiotics (lymecycline). At his 3-month postoperative evaluation, he was reported to have had occasional pustules on the nose, for which he received topical metronidazole.

*Case 7.* A 75-year-old man who had previously experienced positive effects of tetracycline (lymecycline) treatment. Before receiving CO_2_ laser treatment, he had noticed considerable enlargement of sebaceous glands accompanied by a more prominent rhinophyma. No complications were reported (Fig. S5). He received pulse dye laser treatment for residual telangiectasis at the 4-week follow-up.

*Case 8.* A 74-year-old man suffering from moderate rhinophyma was reported to have not responded to oral tetracycline antibiotics (lymecycline) or topical treatments. No immediate complications were reported after receiving laser treatment. At the 3-month follow-up, the healing was described as good. A small annular ulceration, 3x3 mm, on the left side of the nose had yet to heal. Other than that, no complications were reported (Fig. S6).

*Case 9.* A 55-year-old man suffering from moderate rhinophyma who received CO_2_ laser treatment. No complications were reported (Fig. S7).

*Case 10.* A 54-year-old man received laser treatment with no complications. At a 3-month follow-up visit, the nose was completely healed, albeit with moderate scarring (Fig. S8).

*Case 11.* A 66-year-old man suffering from major rhinophyma. He had taken oral isotretinoin 20 mg daily for 4 months until 1 week before receiving the laser treatment. No complications were reported (Fig. S9).

### Adverse events

The only adverse event was reported in Case 3, where the patient received adequate care due to bleeding postoperatively 10 days later. He suffered no life-threatening consequences and completely recovered.

### Questionnaire results

Questionnaires were mailed to the 11 patients with the necessary photo documentation to be included in the study on 26 May 2024. The response rate was 100% (**Table II**). Some 92% (*n*=10) of the patients were very satisfied, with 8 patients giving it the highest possible rating (10/10), 2 patients rating it 9 out of 10, and only 1 patient rating it 7 out of 10 when rating satisfaction with the procedure. Every patient thought the positive effects of the treatment had been sustained. Most importantly, 5 patients in the cohort experienced easier nose breathing after the procedure. None of the patients felt they had experienced any side effects from the procedure. Of the 11 patients, 10 would recommend CO_2_ laser treatment to anyone suffering from rhinophyma. One patient was unsure whether he would recommend the treatment. This individual gave the lowest score (7/10) of all the participants when rating for satisfaction. Although he denied any side effects, he commented that every once in a while he gets bleeding wounds on the nose, and the nose can feel swollen at times.

**Table II:** Results of patient satisfaction survey (11 patients). Numbers in parentheses indicate number of patients

Question	Response % (*n*)
Satisfaction	
0–3	0
4–6	0
7–9	27 (3)
10	73 (8)
Effects maintained	100 (11)
Easier nose-breathing	
Yes	55 (6)
No	45 (5)
Side effects	
None	82 (9)
No comment made	18 (2)
Recommend	
Yes	91 (10)
Not sure	9 (1)
No	

## DISCUSSION

We analysed the long-term results of CO_2_ laser treatment for rhinophyma using photos and patient questionnaires. The initial positive effects of the treatment were sustained for all 11 patients, which is a very positive outcome and supports continuing to treat rhinophyma with CO_2_ laser. None of the patients felt they had experienced any side effects from the treatment, which further supports the previously perceived notion that this treatment option is relatively harmless (1, 5, 10). However, notable hypopigmentation and scarring were seen in some of the patients during both follow-up photos. Hypopigmentation is known to develop up to 6 months post-procedure (5). Considering all our patients were Caucasian of Fitzpatrick skin types I–III, extra caution must be employed when treating skin types IV–VI (5, 11).

More importantly, functional benefits of the procedure were noted for 55% of the patients who reported easier post-procedural nose-breathing. Knowing that severe thickening in the nasal area can obstruct breathing, it is unsurprising that reducing that thickness with a CO_2_ laser can improve nose breathing (12). Facilitated nose breathing has been seen after cosmetic surgery (12); however, evidence to prove this to be the case with other treatment modalities for rhinophyma is lacking. The positive impact on nose breathing should be considered when opting for this treatment option. The more severe the rhinophyma, the more likely there is to be airflow obstruction. In the authors’ opinion, this is due to excessive growth, which can obstruct airflow through the nostrils but can also become too heavy, leading to the nostril collapsing from its original size. It is believed that breathing is facilitated after the excess growth is resected with the laser, as this opens up the nostrils and decreases the weight.

All the patients in this case series were men, therefore whether the same conclusion can be applied to women has yet to be confirmed. However, evidence suggests that CO_2_ laser treatment for rhinophyma in women is a very effective treatment modality (1).

In conclusion, these data further establish CO_2_ laser as a safe and efficient in-office treatment option for moderate to major rhinophyma, as it is associated with long-term maintained results, high patient satisfaction, and few adverse effects (1, 5, 10). When selecting patients for CO_2_ laser treatment, their skin type and any reported obstructed nose breathing should be considered. Patients with difficulty breathing or major rhinophyma should be offered laser treatment early, instead of medical options like isotretinoin, which is less likely to improve breathing. Conversely, patients with darker skin may prefer alternative treatments due to the increased risk of hypopigmentation.

## Supplementary Material

Long-term Satisfaction with CO_2_ Laser Treatment for Moderate to Major Rhinophyma: A Single-centre Study
