# Supplementary material for: Long-term Satisfaction with CO2 Laser Treatment for Moderate to Major Rhinophyma: A Single-centre Study
Source: Acta Derm Venereol. 2025 Jan 3;105:41335. doi: 10.2340/actadv.v105.41335 (PMC11697134; doi:10.2340/actadv.v105.41335)
Supplement: Long-term Satisfaction with CO2 Laser Treatment for Moderate to Major Rhinophyma: A Single-centre Study [file ActaDV-105-41335-s1.pdf]

Supplementary material has been published as submitted. It has not been copyedited, or typeset by Acta Dermato-Venereologica

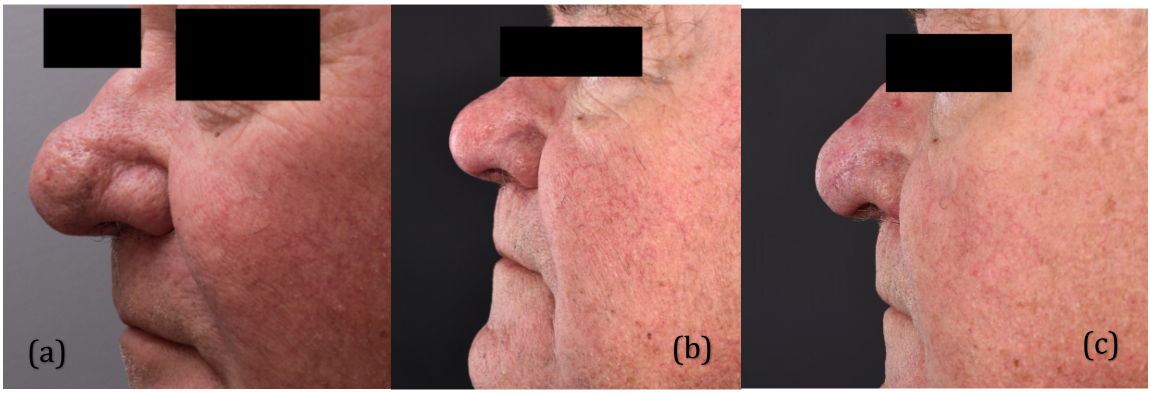

Fig S1. (a) Moderate rhinophyma before undergoing laser treatment (b) 6 weeks afterwards, and (c) 4 years afterwards.

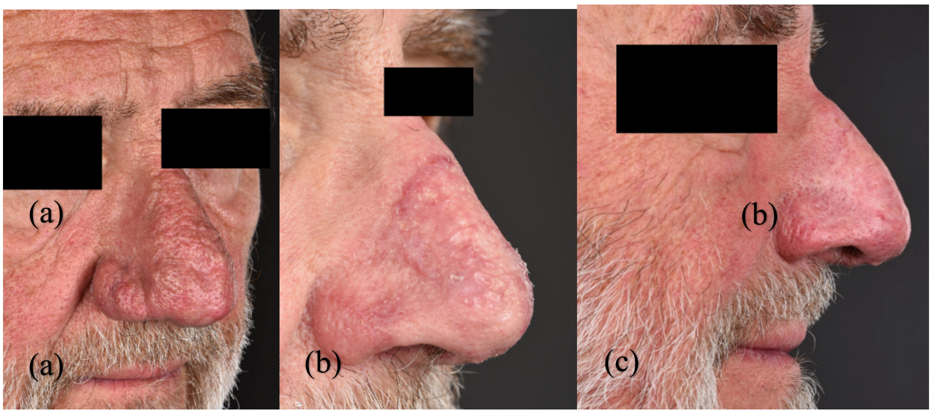

Fig S2. (a) Moderate rhinophyma before receiving laser treatment and (b) 6 weeks post-treatment, revealing minor scarring. (c) 5 years later, the positive effects of the treatment are maintained.

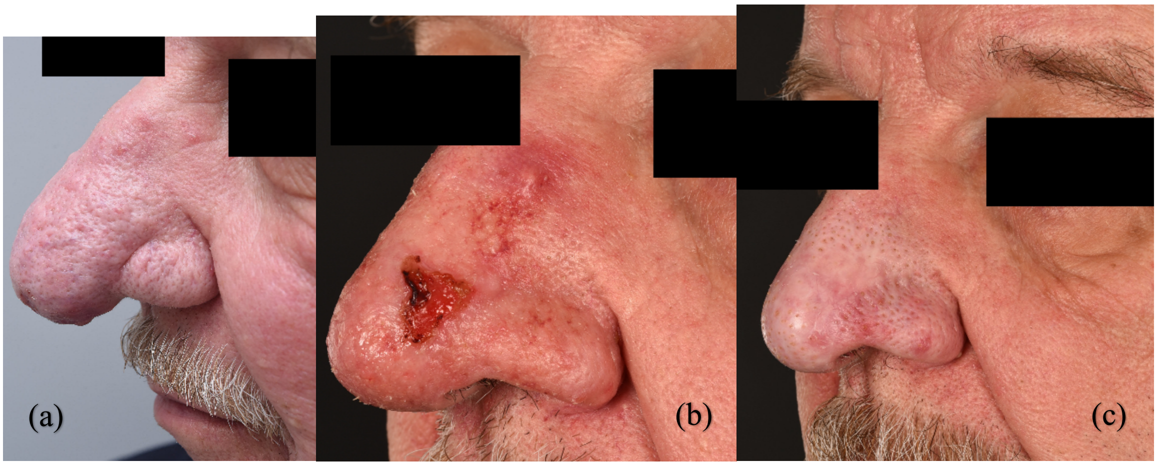

Fig S3. (a) Major rhinophyma before laser treatment with a prominent disfiguration of the nasal tip and (b) 3 weeks post-operatively. (c) 2 years later, slight scarring and hypopigmentation can be seen with otherwise positive results.

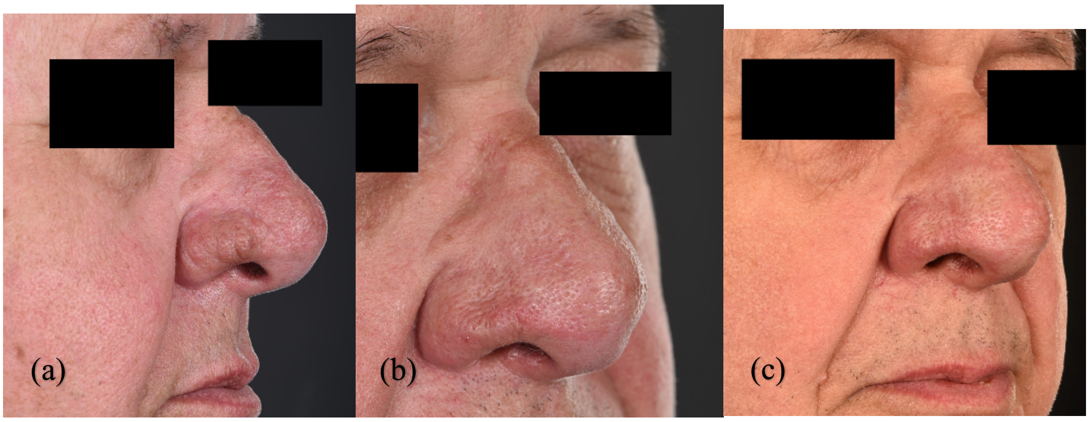

Fig S4. (a) Moderate rhinophyma before receiving laser treatment and (b) 4 months post-operatively, and (c) 6 years later.

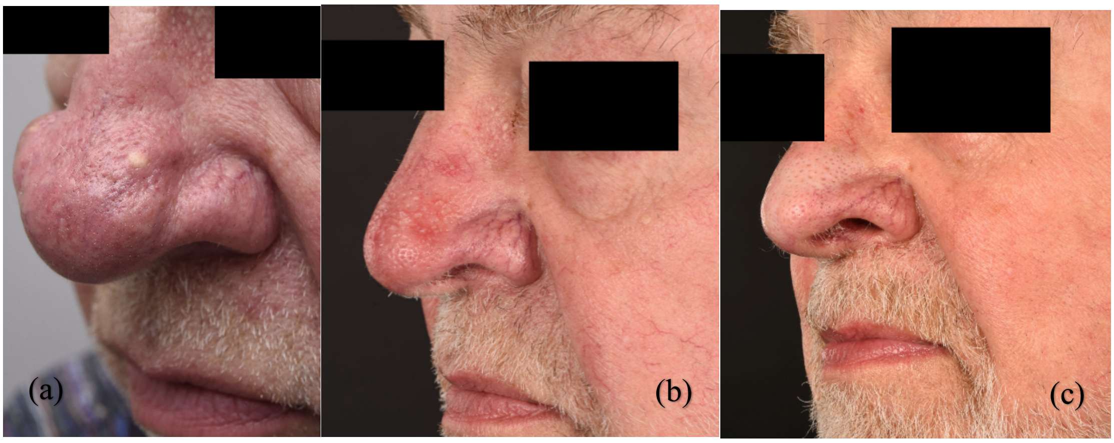

Fig S5. (a) Major rhinophyma before laser treatment and (b) 4 weeks post-operatively. (c) The improved contour of the nose is maintained 3 years later, but slight hypopigmentation and scarring centrally on the nose can be noticed.

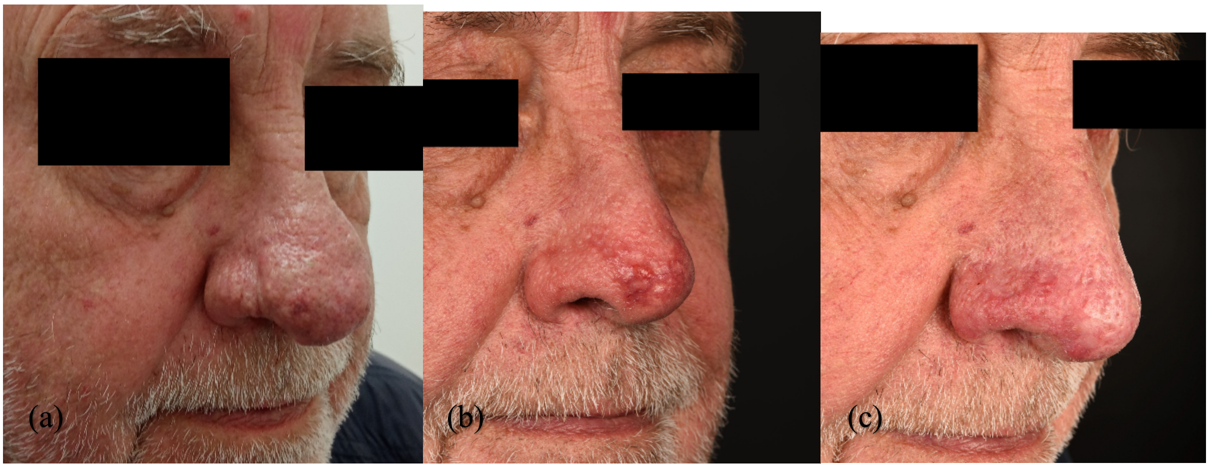

Fig S6. (a) Moderate rhinophyma before laser treatment and (b) 6 weeks later, small ulceration had not yet healed. (c) One year later, the skin had healed well with minimal scaring and a better nose profile.

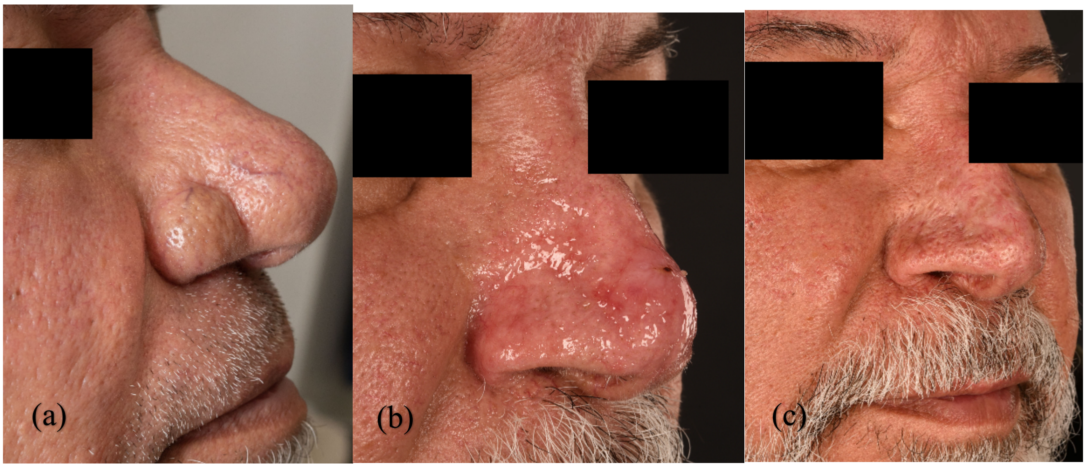

Fig S7. (a) Minor rhinophyma before laser treatment, (b) 6 weeks after (petroleum jelly visible), and (c) 4 months later.

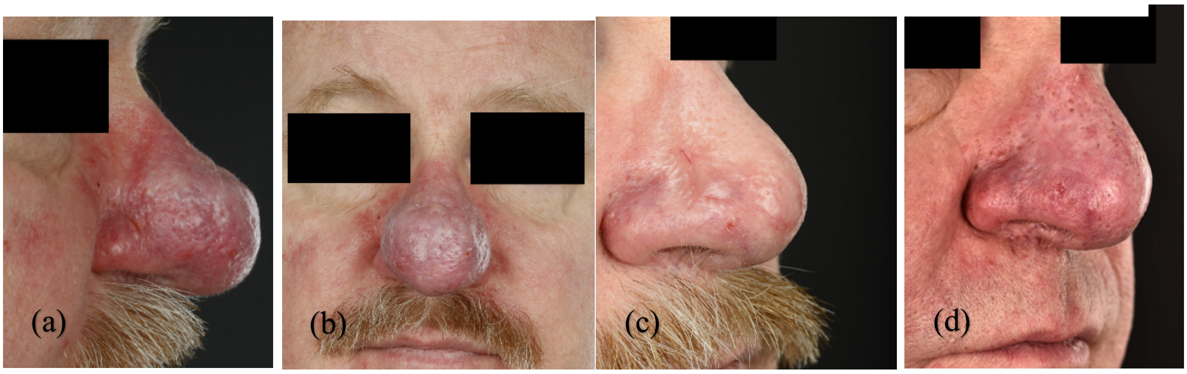

Fig S8. (a,b) Major rhinophyma before laser treatment (c) 4 months later with positive results in a better nose profile. (d) 9 years later, the profile is maintained, however, with some visible scarring and telangiectasis.

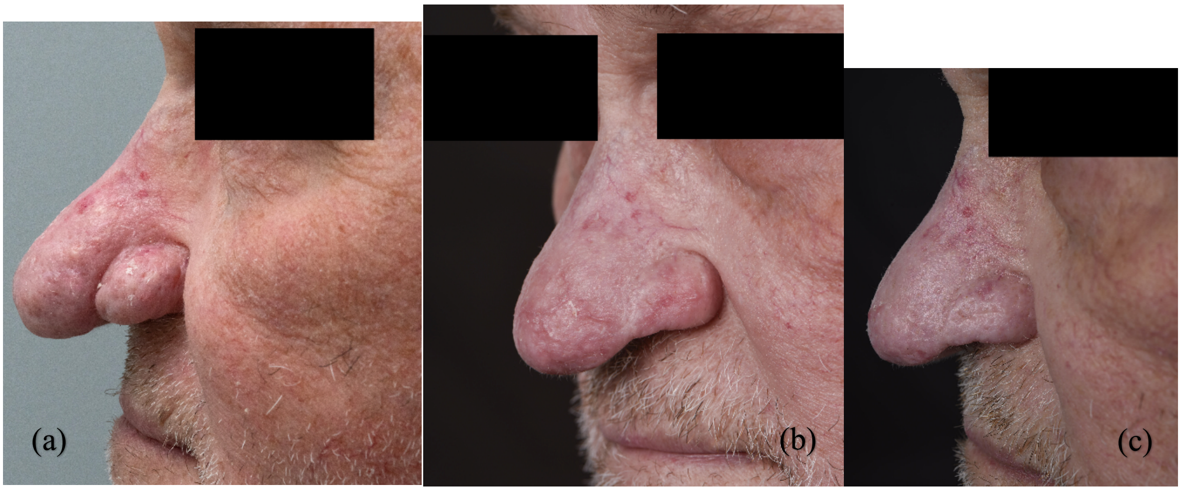

Fig S9. (a) Major rhinophyma before receiving laser treatment and (b) overall better nose profile 2 weeks post-operatively and (c) and 3 months after.
